# Awareness of the diagnosis, treatment, and control of diabetes mellitus in Brazil

**DOI:** 10.11606/s1518-8787.2023057005167

**Published:** 2023-10-24

**Authors:** Luís Antônio Batista Tonaco, Gustavo Velasquez-Melendez, Alexandra Dias Moreira, Flávia Cristina Drumond Andrade, Deborah Carvalho Malta, Mariana Santos Felisbino-Mendes

**Affiliations:** I Universidade Federal de Minas Gerais Escola de Enfermagem Departamento de Enfermagem Materno Infantil e Saúde Pública Belo Horizonte MG Brasil Universidade Federal de Minas Gerais . Escola de Enfermagem . Departamento de Enfermagem Materno Infantil e Saúde Pública . Belo Horizonte , MG , Brasil; II University of Illinois School of Social Work Urbana-Champaign United States University of Illinois . School of Social Work . Urbana-Champaign , United States

**Keywords:** Diabetes Mellitus, Epidemiology, Awareness, Therapeutics

## Abstract

**OBJECTIVE:**

To estimate the proportions of awareness, treatment, and control of diabetes mellitus (DM) in the Brazilian adult population.

**METHOD:**

This is a cross-sectional study, with data from a representative sample of the Brazilian population, taken from the National Health Survey(PNS 2014/2015). Outcomes were defined based on glycated hemoglobin (HbA1c) measurements, self-reported DM diagnosis, and use of hypoglycemic agents or insulin. The proportion of DM awareness, treatment, and control was estimated according to sociodemographic characteristics, health conditions, and access to health services, and their respective 95% confidence intervals.

**RESULTS:**

DM prevalence in the Brazilian population was of 8.6% (95%CI: 7.8–9.3): 68.2% (95%CI: 63.9–72.3) were aware of their diagnosis, 92.2% (95%CI: 88.6–94.7) of those who were aware were undergoing drug treatments, and, of these, 35.8% (95%CI: 30.5–41.6) had controlled HbA1c levels. The proportions of DM awareness, control, and treatment were lower in men aged 18 to 39 years, individuals with low education, without health insurance, and beneficiaries of the *Bolsa Família* program.

**CONCLUSION:**

Approximately one in ten Brazilians has DM. A little more than half of this population is aware of their diagnosis, a condition measured by HbA1c dosage and clinical diagnosis. Among those who know, the vast majority are undergoing drug treatments. However, less than half of these have their HbA1c levels controlled. Worse scenarios were found in subgroups with high social vulnerability.

## INTRODUCTION

Diabetes mellitus (DM) is one of the major public health problems of the twenty-first century. The number of people with DM in the world is estimated at 537 million in 2021, with a projection of 643 million for 2030 and 783 million for 2045 ^1^ . Approximately 50% of DM cases do not receive timely diagnosis ^2^ , and about 90% of cases are type 2 ^1^ . A study conducted with glycated hemoglobin (HbA1c) data obtained from the National Health Survey (PNS) showed a 6.6% prevalence of DM (HbA1c ≥ 6.5%) in the Brazilian population ^3^ . Additionally, self-reported DM increased from 6.2% in 2013 to 7.7% in 2019 ^4^ . In 2021, the *Relatório de Vigilância de Fatores de Risco e Proteção para Doenças Crônicas por Inquérito Telefônico* (Surveillance System for Risk and Protective Factors for Chronic Diseases by Telephone Survey – Vigitel) presented a 9.1% prevalence of self-reported DM in the Brazilian adult population ^5^ . Therefore, estimates of the magnitude of DM in the Brazilian population in general ^3 , 6 , 7^ , and in specific groups, such as men, black and mixed people, people with average or complete educational levels, and obese people, are well defined ^8^ .

Population studies in Latin America showed that the lack of knowledge on the DM diagnosis ranged from 10.3% to 50%, being higher in Guatemala (48.8%), Uruguay (48.7%), and Nicaragua (43.3%), and lower in Colombia (23.5%), South American meridian countries (20.2%), and Costa Rica (10.3–28.4%) ^10^ . The same study also showed that the treatment of this disease in patients ranged from 52.6% to 99%. The prevalence of DM control (HbA1c levels < 7%) ranged from 3.5% to 7.5% ^10^ . However, some studies performed this verification by means of fasting glycemia or casual glycemia and showed a 31.4% to 61.4% variation ^10^ . Specifically in Brazil, a study conducted in the 1990s ^11^ , and, more recently, the *Estudo Longitudinal de Saúde do Adulto* (ELSA) ^6^ identified about 50% of ignorance of the diagnosis.

DM control on a populational level requires an articulation of actions directed to the prevention, detection, and control of the pathology, including a partnership between civil society and government agencies ^7^ . Thus, it is possible to highlight the need to estimate disease control parameters in population subgroups, such as the ability to detect/know the diagnosis, treatment, and control, in addition to its prevalence, as has been discussed internationally ^8 , 9^ .

Inadequate DM control can lead to several complications, such as blindness, chronic kidney disease, and high risk of cardiovascular diseases, and all these outcomes contribute to the increase of health service costs. DM is a manageable disease in primary health care (PHC) services, since public health systems have effective strategies for its early detection, treatment, and control. In Brazil, a study with a regional sample showed worse levels of glycemic control in patients treated by the public health service ^12^ . A study developed in Latin America’s private health services, including the Brazilian health service, showed that blood glucose levels are less controlled in patients with type 2 DM (DM2) ^13^ .

The reliable assessment of the magnitude and treatment of population DM is only possible with representative studies of the Brazilian population and diagnostic techniques of high sensitivity and specificity. Despite the specific data on DM treatment and control in Brazil, the evaluation of these parameters in the population needs to advance. Thus, the aim of this study is to estimate the proportions of awareness, treatment, and control in a representative sample of the Brazilian adult population.

## METHODS

### Study Design and Population

The National Health Survey ( *Pesquisa Nacional de Saúde* – PNS) was conducted in 2013 and extended until 2015 for the collection of biological material. Details about the sampling methodology of the PNS are presented in previous publications ^14^ . This is a survey with cluster sampling in three stages: the census tracts correspond to a fixed number of private households, and for each household a participant aged 18 years or older is randomly selected. The total number of households visited was 81,167. Of these, 69,994 contained residents. At the end, 64,384 home interviews and 60,202 individual interviews were conducted.

The collection of biological material was performed in a subsample with 25% of the census tracts surveyed, totaling 8,952 individuals, who answered the basic questionnaire and were the subjects of our study. To obtain population estimates, the last phase included the weight of post-stratification according to sex, age, education, and region to effectively represent the adult population of the country ^3^ .

Interviews were conducted through the application of a questionnaire, which took place at the participants’ home, by trained interviewers. Sociodemographic data, personal medical history and lifestyle variables were recorded. It also included aspects related to diabetes diagnoses and treatment, measurements of weight, height, waist circumference and blood pressure, in residents aged 18 years or more in each randomly selected household.

### Biological Material Collection

Blood material (7 ml) was collected at any time of the day, without fasting ^14^ . HbA1c was determined from high-performance liquid chromatography (HPLC) of a sample stored in a tube containing ethylenediaminetetraacetic acid (EDTA). It is worth mentioning that the full described step was performed by laboratories certified by the National Glycohemoglobin Standardization Program ^14^ .

### Study Variables

**Figure 1:**
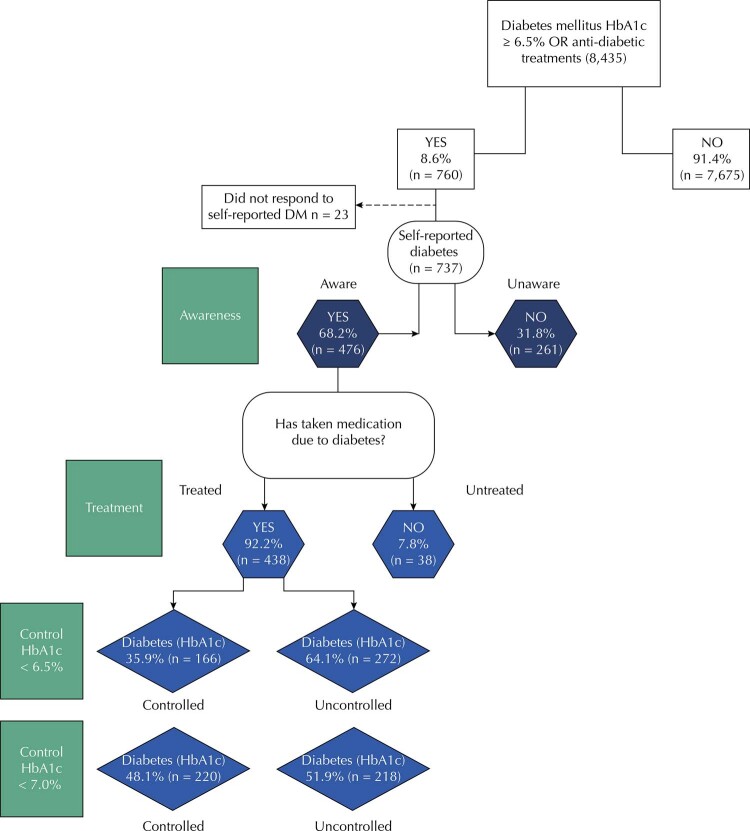
Outcomes of interest: awareness of the diagnosis, treatment, and control of diabetes mellitus.

DM: diabetes mellitus; HbA1c: glycated hemoglobin.

The diabetes diagnosis was defined using HbA1c levels ≥ 6.5% or levels of medication for the disease. Awareness of the diagnosis was defined by the proportion of participants who reported using antidiabetic medication or having received the diagnosis from a health professional. The proportion of participants in treatment was obtained from information on the use of diabetes medications or insulin. The control was defined by two criteria: the proportion of participants who presented HbA1c values < 6.5%, and the proportion of participants with HbA1c < 7% ^2^ ( Figure 1 ). These limits were defined despite the lack of consensus, and recent evidence indicate that the 7% target is related to the prevention of chronic complications ^15^ . This cutoff point should also be further relaxed for individuals at risk of hypoglycemia, such as older adults, being HbA1c < 7.5% in these cases ^15^ .

The sociodemographic variables used were: sex (male and female); age group (18 to 30, 31 to 40, 41 to 50, 51 to 59 and ≥ 60); race/skin color (white, black, yellow/indigenous, and mixed); schooling (no education until complete elementary school, incomplete and complete high school, or incomplete and complete higher education); region of residence (North, Northeast, Southeast, South and Midwest); private health plan (has health insurance or does not have health insurance); receipt of *Bolsa Família* (receives *Bolsa Família* or does not receive *Bolsa Família* ) and self-perceived health (good/excellent, regular, poor, or very poor).

### Data Analysis

The prevalence of DM was calculated according to the diagnostic criteria defined in this study. Next, estimates of the proportion of other outcomes of interest (DM awareness, treatment, and control) and their respective 95% confidence intervals were calculated. These proportions were also estimated according to sociodemographic characteristics, and Pearson’s chi-square test was used to analyze the differences in the proportions of outcomes between the groups. All estimates were calculated in the Stata 14.0 software survey module.

### Ethical Aspects

This study used a secondary, publicly available, and free of charge database, respecting the participants’ confidentiality, not requiring prior approval by the Ethics and Research Committee. The PNS was approved by the National Research Ethics Commission, under CAAE No. 10853812.7.0000.0008, and complies with all ethical precepts, in accordance with the recommendations for research with human beings of Resolution No. 466/12.

## RESULTS

The adult population was composed mostly of women (52.1%), of the white race/skin color (47.8%), with low schooling (49.3%), without health insurance (70.3%), non-beneficiaries of the *Bolsa Família* program (90.6%), with excellent self-perceived health (64.9%), and mainly from the Southeast region (44.3%) ( Table 1 ). DM prevalence of DM was estimated in 8.6% (95%CI: 7.8–9.3) of this population. Most people with DM were female (60.7%), aged over 60 years (54.3%), with low schooling (67.2%), poor self-perceived health (59.5%), and lived in the Southeast region of the country (49.3%).

**Table 1:** Sociodemographic characteristics of the study population and according to the occurrence of diabetes mellitus in a representative sample of the Brazilian population (n = 8,435).

Sociodemographic characteristics	Total		Diabetes				p-value

			Yes		No		
		
	%	95%CI	% ^a^	95%CI	% ^a^	95%CI	
Sex							0.0001
Male	47.8	46.4–49.3	39.4	35.0–43.9	48.7	47.1–50.2	
Female	52.2	50.7–53.6	60.7	56.1–56.0	51.4	49.8–52.9	
Age (years)							< 0.0001
18–39	41.1	39.7–42.6	09.4	06.8–12.8	44.1	42.6–45.7	
40–49	19.7	18.6–20.8	12.8	10.0–16.2	20.3	19.2–21.5	
50–59	17.0	15.9–17.9	23.6	20.1–27.6	16.3	15.5–17.4	
≥ 60	22.2	21.2–23.4	54.3	49.7–58.7	19.2	18.2–20.4	
Race/Skin Color							0.288
White	47.8	46.4–49.3	48.4	43.9–53.0	47.8	46.2–49.3	
Black	9.3	8.5–10.2	11.3	08.7–14.7	09.2	08.3–10.1	
Yellow and indigenous	0.9	0.7–1.3	0.9	00.5–01.5	01.0	00.7–01.3	
Brown	42.0	40.5–43.3	39.4	35.2–43.6	42.1	40.7–43.6	
Education level							< 0.0001
No education to complete primary education	49.3	47.8–50.7	67.2	62.7–71.5	47.6	46.1–49.1	
Incomplete and complete secondary education	33.8	32.4–35.2	23.3	19.4–27.6	34.8	33.3–36.3	
Incomplete and complete higher education	16.9	15.8–18.1	09.5	07.2–12.5	17.7	16.4–18.9	
Health insurance							0.586
Yes	29.7	28.3–31.1	28.6	24.6–33.0	29.8	28.4–31.3	
No	70.3	68.9–71.6	71.4	67.1–75.4	70.2	68.7–71.6	
*Bolsa Família*							0.427
Yes	9.4	8.7–10.1	08.4	06.1–11.4	09.5	08.8–10.3	
No	90.6	89.8–91.3	91.6	88.6–93.9	90.5	89.7–91.2	
Self-perceived health							< 0.0001
Good and excellent	64.9	63.6–62.2	36.0	31.8–40.5	67.6	66.3–68.9	
Poor, very poor, and regular	35.1	33.8–36.4	64.0	59.5–68.2	32.4	31.1–33.8	
Region							0.019
North	6.9	6.6–7.3	05.1	04.3–06.2	07.1	06.7–07.5	
Northeast	26.3	25.2–27.3	23.8	20.9–27.0	26.5	25.4–27.6	
Southeast	44.3	42.8–45.8	49.3	44.8–53.8	43.8	42.2–45.5	
South	15.0	14.1–15.6	13.4	10.9–16.4	15.1	14.2–16.2	
Midwest	7.5	7.0–8.0	08.3	06.8–10.2	07.4	06.9–08.8	

95%CI: 95% confidence interval.

^a^ Pearson’s chi-square test.

**Figure 2:**
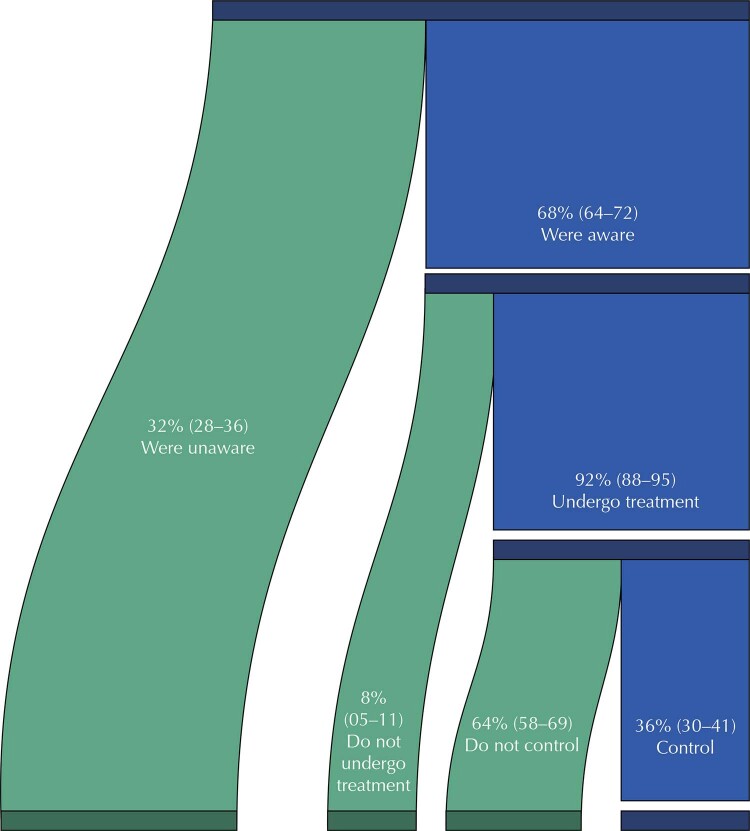
Prevalence and 95% confidence intervals of awareness of the diagnosis, treatment, and control of diabetes mellitus in the Brazilian population.

Note: Awareness of the diagnosis was defined by the proportion of participants who reported using antidiabetic medication or having received the diagnosis from a health professional. The proportion of participants in treatment was obtained from information on the use of diabetes medications or insulin. Control was defined by the proportion of participants who had glycated hemoglobin (HbA1c) values lower than 6.5%.

We estimated that 68.2% (95%CI: 63.9–72.3) of people with diabetes were aware of their diagnosis and, of these, 92.2% (95%CI: 88.6–94.7) were on medication treatments. We estimated that 35.9% (95%CI: 30.5–41.6) of people had HbA1c levels considered normal (< 6.5%) and 48.1% (95%CI: 42.2–53.9) had HbA1c levels below 7%, that is, with normalized or controlled glucose homeostasis, among those who received some type of pharmacological treatment ( Figure 2 ).

The proportion of DM awareness was of 41.7% (95%CI: 25.6–60.0) in 18 to 39 years age group, 65.7% (95%CI: 60.6–70.5) among participants without health insurance, 47.5% (95%CI: 31.6–64.0) in the group which received *Bolsa Família* , 51.8% (95%CI: 44.0–56.0) among those with good/excellent self-perceived health, and 51.5% (95%CI: 42.9–60.0) in residents of the North region of the country ( Table 2 ). The same table shows that the proportion of drug treatment was lower in males (88%) (95%CI: 80.0–93.1), those among the 40 to 49 years age group (84.6%) (95%CI: 61.6–94.9), and those who did not have health insurance (89.9%) (95%CI: 85.0–93.4).

**Table 2:** Proportion of diabetes mellitus awareness, treatment, and control in the Brazilian adult population according to sociodemographic variables (n = 8,435).

Sociodemographic characteristics	Diabetes mellitus awareness			Diabetes mellitus treatment			HbA1c control < 6.5%			HbA1c control < 7.0%		
			
	%	95%CI	p ^a^ value	%	95%CI	p ^a^ value	%	95%CI	p ^a^ value	%	95%CI	p ^a^ value
Total	68.2	63.9–72.3		92.2	88.6–94.7		35.9	30.5–41.6		48.1	42.2–53.9	
Sex			0.84			0.268			0.98			0.271
Male	67.6	60.6–74.0		88.0	80.0–93.1		35.7	26.8–45.7		43.6	33.9–53.8	
Female	68.6	63.0–73.7		94.7	91.3–96.8		35.9	29.4–42.9		50.6	43.5–57.7	
Age (years)			< 0.0001			0.421			0.005			0.002
18–39	41.7	25.6–60.0		94.0	76.9–98.7		14.2	05.4–32.7		14.2	05.4–32.7	
40–49	52.1	39.1–64.9		84.6	61.6–94.9		28.4	14.4–48.1		48.1	0.29–0.68	
50–59	71.6	63.1–78.8		92.5	84.7–96.5		25.3	16.3–37.1		38.3	0.26–0.50	
≥ 60	74.8	69.3–79.6		93.0	88.8–96.5		42.2	36.2–50.5		55.1	0.42–0.62	
Race/Skin Color			0.117			0.919			0.7132			0.658
White	72.7	66.0–78.4		92.2	86.9–92.3		38.0	30.1–46.6		50.6	42.1–59.1	
Black	60.8	46.3–73.6		90.9	76.2–96.9		34.0	18.7–53.4		48.3	30.1–66.9	
Yellow and indigenous	48.2	23.1–74.3		100.0	–		40.3	12.1–76.8		40.3	12.1–76.8	
Brown	65.3	59.1–71.1		91.3	85.8–94.8		33.2	25.9–41.4		44.5	36.2–53.1	
Education level			0.668			0.547			0.735			0.848
No education to complete primary education	68.2	63.0–73.0		91.1	86.3–94.3		35.3	29.1–42.1		48.9	42.2–55.7	
Incomplete and complete secondary education	66.1	56.1–74.8		93.9	85.1–97.6		34.2	22.6–48.1		44.8	31.7–58.6	
Incomplete and complete higher education	73.4	59.8–83.6		95.5	82.2–99.0		42.4	26.4–60.2		49.6	32.1–67.3	
Health insurance			0.079			0.015			0.976			0.79
Yes	74.3	65.9–81.2		96.9	92.3–98.8		35.7	26.7–45.8		46.9	36.8–57.2	
No	65.7	60.6–70.5		89.9	85.0–93.4		35.9	29.4–42.9		48.6	41.6–55.7	
*Bolsa Família*			0.006			0.221			0.028			0.41
Yes	47.5	31.6–64.0		96.8	85.9–99.3		17.6	07.9–34.5		38.5	19.3–62.1	
No	70.1	65.7–74.2		91.9	88.1–94.5		37.0	31.4–43.0		48.7	42.7–54.7	
Self-perceived health			< 0.0001			0.364			0.002			0.0077
Good and excellent	51.8	44.0–56.0		90.0	82.6–94.5		50.8	39.3–62.1		61.6	49.7–72.2	
Poor, very poor, and regular	77.4	72.7–81.5		93.0	88.5–95.8		30.4	24.7–36.9		43.1	36.6–49.5	
Region			0.004			0.864			0.71			0.9052
North	51.5	42.9–60.0		92.8	83.6–97.0		37.3	26.1–49.9		47.1	35.1–59.5	
Northeast	59.2	52.7–65.4		92.1	86.6–95.5		40.0	32.0–48.6		50.8	42.3–59.3	
Southeast	73.8	66.2–80.2		92.9	86.4–96.4		35.7	27.0–44.7		47.5	38.3–56.9	
South	67.2	56.1–76.7		90.9	79.5–96.2		30.0	19.0–43.9		44.5	31.4–58.4	
Midwest	71.3	60.9–79.8		89.1	78.6–94.8		37.1	25.9–49.8		50.5	37.7–63.2	

95%CI: 95% confidence interval.

^a^ Pearson’s chi-square test.

Table 2 also shows the proportions of DM patients who are under control using two cutoff points: HbA1c < 6.5% was 35.8%, and HbA1c < 7% was 48.1%. There was also a lower control, from the first cutoff point, in males (35.7%) (95%CI: 26.8–45.7) and those who reported poor self-perceived health (30.4%) (95%CI: 24.7–36.9). Regarding the second cutoff point, a lower control was observed in males (43.6%) (95%CI: 33.9–53.8), in those with incomplete/complete secondary education (44.8%) (95%CI: 31.7–58.6), and those who reported poor self-perceived health (43.1%) (95%CI: 36.6–49.5).

## DISCUSSION

In this study, we estimate that approximately one in ten Brazilians has a diabetes diagnosis and, of these, 68.2% are aware of their diagnosis. In addition, most diabetics are undergoing drug treatment, and less than half of these have their HbA1c levels below 6.5%, that is, controlled. When considering more flexible glycemic control goals,we observed that 48.1% of the adult population has HbA1c < 7%, and 65.7% of older adults have HbA1c < 7.5%.

The highest DM prevalence was in the group over 60 years of age, who declared themselves to be of the yellow/indigenous race/skin color, with low schooling, with poor self-perceived health, and in Brazil’s Midwest and Southeast populations. The proportions of DM awareness, control, and treatment were lower in men aged 18 to 39 years, individuals with low education, without health insurance, and beneficiaries of the *Bolsa Família* program.

The high proportions of participants who are unaware of their diagnosis are, in this study, concentrated in groups of low socioeconomic levels, participants of the B *olsa Família* program, and residents of the northern region of the country, as another study already observed ^16^ .

An additional aspect, which was shown in a recent study, is that populations with few socioeconomic resources also have high rates of smoking, overweight and obesity prevalence, low consumption of fruits and vegetables, and high consumption of sugar-sweetened beverages, and, in women, low access to cervical and breast cancer screening programs ^16^ .

Strategies, such as the National Campaign for DM Detection, which reached more than 20 million Unified Health System (SUS) users aged over 40 years ^17^ , should be reissued. However, strategies for the use of light care, low cost and wide accessibility technology for the Brazilian population may be necessary.

The Brazilian population is certainly not far from global prevalence estimates. A recent study shows that approximately 50% of adult diabetics are not aware of their diagnosis, and that, of these, 84.3% are living in developing countries ^18^ . A low-cost, non-invasive, and easy-to-apply alternative would be the use of the Finnish Diabetes Risk Score (FINDRISC), which measures the risk of DM2 development in adults. This strategy has had its validity demonstrated at a national level ^19^ .

Low prevalence of controlled diabetes, measured by glycated hemoglobin levels (< 6.5%), was present in the low schooling and beneficiaries of the *Bolsa Família* program groups. These findings reinforce the link between social vulnerability and low access to effective disease control, corroborating findings of other studies ^20^ .

Estimates using data from the PNS 2019 showed that younger people had lower medication consumption ^3^ , which can be justified by the lower severity of the pathology, and it is also possible to favor management through non-pharmacological measures, such as encouraging physical activity and healthy eating. In addition, type 1 DM (DM1), not identified in this study, may present lower glycemic control, due to the severity of the disease, as well as its resistance to medication use ^21^ .

We were also able to identify a higher frequency of hospitalization in young people aged 18 to 29 years, which is understandable given the higher prevalence of DM1 in young adults, the acute symptoms of the disease and the non-adaptation to new care and lower adherence to caregiver practices ^4^ . Lower hospitalization due to DM or complications were identified among women, which may mean better disease control among them, which coincides with our findings ^4^ .

The high proportion of people with diabetes in treatment estimated in this study refers to the public policies implemented to improve access to medication, through the Popular Pharmacy ( *Farmácia Popular* ) and Health Has No Price ( *Saúde Não Tem Preço* ) programs, which allow free access to these drugs in Basic Health Units and pharmacies accredited to the programs. The surveillance systems also showed that 70.3% of people diagnosed with DM obtained free oral medicines through the SUS pharmacy or the Popular Pharmacy ( *Farmácia Popular* ) program, and that 90% had free access to insulin ^22^ . Notably, the National Survey on Access, Use and Promotion of the Rational Use of Medicines ( *Pesquisa Nacional sobre Acesso, Utilização e Promoção do Uso Racional de Medicamentos* – PNAUM), a population-based household survey, showed that 21.4% of participants paid for diabetes medication, and 78.6% got it for free ^23^ . Still confirming these conclusions, 57.4% of the PNS 2013 participants reported obtaining drugs for diabetes via the *Farmácia Popular* program ^24^ . In this study, we do not have information to clarify whether the prevalence of participants without treatment is related to the choice of medications or to access difficulties ^25^ .

In PHC, the follow-up of patients with diabetes after diagnosis includes medical and nursing consultations, participation in diabetic groups. The number of consultations with each professional changes according to the patient’s clinical condition ^26^ . It is important to emphasize, for those who are under glycemic control, the need to perform fasting glucose and HbA1c tests twice a year and, for those who are not, every three months ^26^ .

The maintenance of glucose levels within the normal range is essential for coping with DM. In this study, the prevalence of control was low, not far from findings in other populations, such as those of Iranian Kurdistan (18.30%) ^27^ , Korea (42.5%) ^28^ , and Myanmar (40.8%) ^29^ . Previous studies estimated a prevalence of 26% and 78% of glycemic level control in a population attended by public health services and in a population attended by private services, respectively, but these are local studies without national representation, which makes it difficult to compare them to our study ^12 , 13^ .

Failures in glycemic control may contribute to increase risks of cardiovascular diseases, nephropathies, neuropathies, retinopathies, and hospitalizations ^30^ . It is noteworthy that the complexity inherent to drug therapies contributes to increase medication error risks and requires the user to have the skills to comply with the care provided by health professionals ^31 , 32^ . The challenge of drug therapy was accompanied by the quantity of drugs in use, resulting from patients with high complexity, who used polypharmacy ^33^ . The patient’s empowerment in the self-care process, health education, especially in relation to medication schedule information, and the correct use of drugs in accordance with the medical prescription, are necessary strategies to achieve disease control ^34^ .

Glycemic control is essential to decrease the risk of DM complications and cardiovascular diseases. Other important factors are the lipid profile monitoring and the appropriate treatment to achieve glycemic control. DM management in primary care follows a strategy of healthy lifestyle habit encouragement. However, in practice, the program is mostly focused on medicine supply. This work process hinders the achievement of objectives such as the recognition and dimensioning of health problems, both individually and collectively, which help more effectively in health actions/interventions, as shown in a study developed in the city of São Paulo ^35^ . Studies show that there is no link between patients with DM and the actions of health professionals, corroborating for the discontinuity of treatment adherence and directly impacting its control ^36^ .

The low DM control results identified in this study can be explained by the complexity of the management of the disease, which includes the monitoring of glycemic values, adherence to treatment and the inclusion of regular physical activity and diet changes ^37^ . In this perspective, the strategies that contribute to the patient’s empowerment can help in the process of developing the adoption of new attitudes and skills, which will promote changes in habits/lifestyle and, consequently, in self-care. Clinical trials using education strategies through telephone interventions, training programs and home visits, have shown that these interventions contribute positively to an improvement of HbA1c levels results, to empowerment and self-care ^38^ . Home visits by community health agents (CHA) contribute to controlling and supervising treatment and improving adherence to self-care practices related to DM2 ^39^ .

Notably, the magnitude of harm shown may worsen as a result of the new PHC financing model in SUS ^40^ , called Prevent Brazil ( *Previne Brasil* ), because it only strengthens biomedical strategies, which could lead to delays in DM detection and worsen and decrease control prevalence. In addition, it is worth pointing out that the effects generated by the covid-19 pandemic may, in the short term, affect the performance of care provided to chronic diseases, worsening control and detection levels of the disease ^41^ .

A limitation of this study is the lack of data regarding the medication used, its time of use, and the adherence to treatment, which was limited to recording only the use of antidiabetic drugs and/or insulin injections. Non-pharmacological measures are also necessary for glycemic control, and they were not evaluated in this study. These data would allow a better understanding of the low performance of glycemic level control found in the Brazilian population, despite the wide access to medication and treatment. However, the use of population-based data and national representativeness constitutes adequate external validity.

We highlight that the measurement of HbA1c levels collected from venous and non-capillary blood was used, which is considered the gold standard for detecting the disease. The lack of consensus to define the cutoff point of HbA1c in disease control hinders the process of evaluation and monitoring of DM. Therefore, this study chose to work with two cutoff points.

Another limitation is the non-distinction between the DM types (DM1 or DM2). This is an unprecedented population study that uses laboratory data with representativeness of the Brazilian population to estimate DM awareness, treatment, and control, which are fundamental aspects to help public health programs cope with the disease.

The proportion of DM awareness in the Brazilian population was estimated at 68%, and of these, 92% were undergoing drug treatment. Adequate control was estimated at 36%, considering the strictest criterion (HbA1c < 6.5%). Some population subgroups, such as those who do not have health insurance, those who reported having poor self-perceived health, and beneficiaries of the *Bolsa Família* program, presented worse performance regarding awareness of the disease. The data from this study may contribute to strengthen public policies that aim to promote healthy lifestyles. The implementation of innovative strategies to assist in DM control is fundamental to face the disease burden attributed to DM in Brazil.
