# Supplementary material for: Awareness of the diagnosis, treatment, and control of diabetes mellitus in Brazil
Source: Rev Saude Publica. 2023 Oct 24;57:75. doi: 10.11606/s1518-8787.2023057005167 (PMC10609647; doi:10.11606/s1518-8787.2023057005167)
Supplement: Translation

# Translation: Conhecimento do diagnóstico, tratamento e controle do diabetes mellitus no Brasil

**Authors:** Luís Antônio Batista Tonaco, Gustavo Velasquez-Melendez, Alexandra Dias Moreira, Flávia Cristina Drumond Andrade, Deborah Carvalho Malta, Mariana Santos Felisbino-Mendes

**Affiliations:** I Brasil Universidade Federal de Minas Gerais. Escola de Enfermagem. Departamento de Enfermagem Materno Infantil e Saúde Pública. Belo Horizonte, MG, Brasil; II United States University of Illinois. School of Social Work. Urbana-Champaign, United States

**Keywords:** Diabetes Mellitus, Epidemiologia, Conscientização, Terapêutica

## Abstract

**OBJETIVO:**

Estimar as proporções dos indivíduos que têm conhecimento do diagnóstico, tratamento e controle do diabetes mellitus (DM) na população adulta brasileira.

**MÉTODO:**

Este é um estudo transversal, com dados de amostra representativa da população brasileira, provenientes da Pesquisa Nacional de Saúde (PNS 2014/2015). Os desfechos foram definidos com base na medida de hemoglobina glicada (HbA1c), no diagnóstico autorreferido de DM e no uso de hipoglicemiantes ou de insulina. Estimou–se a proporção do conhecimento, tratamento e controle do DM de acordo com as características sociodemográficas, condição de saúde e de acesso aos serviços de saúde, e seus respectivos intervalos de 95% de confiança (IC95%).

**RESULTADOS:**

A prevalência de DM na população brasileira foi 8,6% (IC95% 7,8–9,3), 68,2% (IC95% 63,9–72,3) tinham conhecimento do seu diagnóstico, 92,2% (IC95% 88,6–94,7) dos que tinham conhecimento realizam tratamento medicamentoso, e desses, 35,8% (IC95% 30,5–41,6) tinham os níveis de HbA1c controlados. As proporções de conhecimento, controle e tratamento foram menores nos homens, com idade de 18 a 39 anos, indivíduos que possuem baixa escolaridade, sem plano de saúde e beneficiários do Programa Bolsa Família.

**CONCLUSÃO:**

Aproximadamente um em cada dez brasileiros apresenta DM. Um pouco mais da metade desta população tem conhecimento do seu diagnóstico, condição aferida por dosagem de HbA1c e diagnóstico clínico. Entre os que sabem, a grande maioria está sob tratamento medicamentoso. Porém, menos da metade destes tem seus níveis de HbA1c controlados. Cenários piores foram encontrados em subgrupos com alta vulnerabilidade social.

## INTRODUÇÃO

O diabetes mellitus (DM) constitui-se um dos maiores problemas de saúde pública do século XXI. Estima-se que o número de pessoas com DM no mundo era de 537 milhões em 2021, com projeção para 643 milhões em 2030 e 783 milhões em 2045^1^. Aproximadamente 50% dos casos de DM não recebem diagnóstico oportuno^2^, e cerca de 90% dos casos desta doença são do tipo 2^1^. Estudo realizado com dados da hemoglobina glicada (HbA1c), obtidos da Pesquisa Nacional de Saúde (PNS), mostrou uma prevalência de 6,6% de DM (HbA1c ≥ 6,5%) na população brasileira^3^. Adicionalmente, o DM autorreferido teve um aumento de 6,2%, em 2013, para 7,7% em 2019^4^. O Relatório de Vigilância de Fatores de Risco e Proteção para Doenças Crônicas por Inquérito Telefônico (Vigitel) apresentou, em 2021, uma prevalência de DM autorreferido de 9,1% na população adulta brasileira^5^. Portanto, estimativas da magnitude do DM na população brasileira em geral^3 , 6 , 7^, e em grupos específicos, tais como homens, pretos e pardos, com nível de escolaridade médio ou completo e obesos, estão bem delimitadas^8^.

Estudos populacionais na América Latina mostraram que a ausência de conhecimento do diagnóstico de DM variou de 10,3% a 50%, sendo maior na Guatemala (48,8%), Uruguai (48,7%) e Nicarágua (43,3%), e menor na Colômbia (23,5%), países do meridiano da América do Sul (20,2%) e na Costa Rica (10,3–28,4%)^10^. Nesse mesmo estudo também foi mostrado que o tratamento dessa doença nos pacientes variou de 52,6% a 99%. A prevalência do controle de DM (níveis de HbA1c < 7%) variou de 3,5% a 7,5%^10^. Porém, alguns estudos realizaram essa verificação por meio da glicemia de jejum ou pela glicemia casual, e mostraram variação de 31,4% a 61,4%^10^. Especificamente no Brasil, um estudo realizado na década de 1990^11^, e, mais recentemente, o estudo Estudo Longitudinal de Saúde do Adulto (ELSA)^6^, identificaram cerca de 50% de desconhecimento do diagnóstico.

O controle do DM em nível populacional exige uma articulação de ações direcionadas para prevenção, detecção e controle da patologia, incluindo parceria da sociedade civil com órgãos governamentais^7^. Assim, destaca-se a necessidade de, além da sua prevalência, estimar parâmetros do controle da doença em subgrupos populacionais, tais como a capacidade de detecção/conhecimento do diagnóstico, tratamento e controle, como vem sendo discutido em âmbito internacional^8 , 9^.

O controle inadequado do DM pode levar a diversas complicações, como cegueira, doença renal crônica e alto risco de doenças cardiovasculares, e todos esses desfechos contribuem com o alto custo para os serviços de saúde. O DM é uma doença passível de manejo nos serviços de atenção primária à saúde (APS), uma vez que os sistemas públicos de saúde têm estratégias eficazes para sua detecção precoce, tratamento e controle. No Brasil, um estudo com amostra regional mostrou níveis piores de controle glicêmico de pacientes tratados no serviço público de saúde^12^. Estudo desenvolvido em serviço privado de saúde da América Latina, incluindo o Brasil, evidenciou que os níveis de glicemia estão subcontrolados em pacientes com DM tipo 2 (DM2)^13^.

A avaliação confiável da magnitude e do tratamento do DM populacional só é possível com estudos representativos da população brasileira e técnicas de diagnóstico de alta sensibilidade e especificidade. Embora existam alguns dados específicos sobre o tratamento e controle da DM no Brasil, é preciso avançar na avaliação desses parâmetros na população. Assim, o objetivo deste estudo é estimar as proporções de conhecimento do diagnóstico, tratamento e controle do DM em uma amostra representativa da população adulta brasileira.

## MÉTODOS

### Desenho e População do Estudo

A PNS foi conduzida em 2013 e estendida até 2015 para coleta de material biológico. Detalhes sobre a metodologia de amostragem da PNS estão apresentados em publicações anteriores^14^. Resumidamente, trata-se de inquérito com amostragem por conglomerados em três estágios: os setores censitários correspondem a um número fixo de domicílio particular, e para cada domicílio foi sorteado um participante com idade igual ou superior a 18 anos. O número total de domicílios visitados foi de 81.167. Destes, 69.994 continham residentes. Ao final, foram realizadas 64.384 entrevistas domiciliares e 60.202 individuais.

A coleta de material biológico foi realizada em subamostra com 25% dos setores censitários pesquisados, totalizando 8.952 indivíduos, que responderam ao questionário base e foram os sujeitos do presente estudo. Com o propósito de obter estimativas populacionais, a última fase contou com o peso de pós-estratificação de acordo com sexo, idade, escolaridade e região, a fim de alcançar representatividade da população adulta do país^3^.

Foram realizadas entrevistas por meio da aplicação de um questionário no domicílio do participante por entrevistadores treinados. Dados sociodemográficos, histórico médica pessoal e variáveis de estilo de vida foram registrados. Também incluiu aspectos relacionados a diagnósticos e tratamento de diabetes, aferições de peso, altura, circunferência da cintura e pressão arterial no morador com 18 anos ou mais em cada domicílio selecionado aleatoriamente.

### Coleta de Material Biológico

A coleta de material sanguíneo (7 ml), foi feita em qualquer horário do dia, sem realização de jejum^14^. A HbA1c foi determinada a partir da cromatografia líquida de alta performance (HPLC) de amostra armazenada em tubo contendo ácido etilenodiamino tetra-acético (EDTA). Vale mencionar que toda a etapa descrita foi executada por laboratórios certificados pelo *National Glycohemoglobin Standardization Program* ^14^.

### Variáveis de Estudo

O diagnóstico de diabetes foi definido usando os níveis de HbA1c ≥ 6,5%, ou de medicação para a doença. O conhecimento do diagnóstico foi definido pela proporção de participantes que afirmaram terem recebido o diagnóstico de um profissional de saúde ou que declararam utilizar medicação antidiabética. A proporção de participantes em tratamento foi obtida a partir da informação sobre o uso de medicamentos para diabetes ou insulina. O controle foi definido por dois critérios: a proporção de participantes que apresentaram valores de HbA1c < 6,5%, e a proporção de participantes com HbA1c < 7%^2^( Figura 1 ). Esses limites foram definidos a partir da falta de consenso e de evidências mais recentes indicarem que a meta de 7% está relacionada à prevenção de complicações crônicas^15^. Esse ponto de corte deve, inclusive, ser flexibilizado ainda mais para indivíduos com risco de hipoglicemias, como os idosos, o qual é HbA1c < 7,5%^15^.

**Figura 1:** Desfechos de interesse: conhecimento do diagnóstico, tratamento e controle do diabetes mellitus.

DM: diabetes mellitus; HbA1c: hemoglobina glicada.

As variáveis sociodemográficas utilizadas foram: “sexo” (masculino e feminino), “faixa etária” (18 a 30, 31 a 40, 41 a 50, 51 a 59 e ≥ 60), “raça/cor” (branca, preta, amarela/indígena e parda), “escolaridade” (sem instrução até fundamental completo, ensino médio incompleto e médio completo, e ensino superior incompleto e superior completo), “região de residência” (Norte, Nordeste, Sudeste, Sul e Centro–Oeste), além das variáveis “plano de saúde privado” (tem plano de saúde e não tem plano de saúde), “recebimento de Bolsa Família” (recebe Bolsa Família e não recebe Bolsa Família) e “autopercepção de saúde” (boa/ótima, regular, ruim e muito ruim).

### Análise de Dados

Foi calculada a prevalência de DM conforme o critério de diagnóstico definido neste estudo. Em seguida foram calculadas as estimativas de proporção dos demais desfechos de interesse (conhecimento do diagnóstico, tratamento e controle do DM) e seus respectivos intervalos de 95% de confiança. Essas proporções também foram estimadas segundo as características sociodemográficas, e utilizou-se o teste qui-quadrado de Pearson para a análise das diferenças das proporções dos desfechos entre os grupos. Todas as estimativas foram calculadas no módulo *survey* , do software Stata 14.0.

### Aspectos Éticos

Para este estudo foi utilizada base de dados secundária, de acesso público e gratuito, respeitando a confidencialidade dos participantes, dispensando aprovação prévia em Comitê de Ética e Pesquisa. Ressalta-se que a PNS foi aprovada pela Comissão Nacional de Ética em Pesquisa com respectivo parecer: CAAE nº 10853812.7.0000.0008, e cumpre todos os preceitos éticos, em conformidade com as recomendações para pesquisa com seres humanos da Resolução nº 466/12.

## RESULTADOS

A população adulta foi composta em sua maioria por mulheres (52,1%), de raça/cor branca (47,8%), com baixa escolaridade (49,3%), sem plano de saúde (70,3%), não beneficiários do Programa Bolsa Família (90,6%), com ótima percepção de saúde (64,9%), e principalmente provenientes da região Sudeste (44,3%) ( Tabela 1 ). A prevalência de DM foi estimada em 8,6% (IC95% 7,8–9,3) desta população. As pessoas com DM eram, em sua maioria, do sexo feminino (60,7%), com idade superior a 60 anos (54,3%), baixa escolaridade (67,2%), percepção de saúde ruim (59,5%) e residiam na região Sudeste do país (49,3%).

**Tabela 1:** Características sociodemográficas da população de estudo e segundo a ocorrência de diabetes mellitus em amostra representativa da população brasileira (n = 8.435).

Características sociodemográficas	Total		Diabetes				valor de p

			Sim		Não		
		
	%	IC95%	%^a^	IC95%	%^a^	IC95%	
Sexo							0,0001
Masculino	47,8	46,4–49,3	39,4	35,0–43,9	48,7	47,1–50,2	
Feminino	52,2	50,7–53,6	60,7	56,1–56,0	51,4	49,8–52,9	
Idade (anos)							< 0,0001
18–39	41,1	39,7–42,6	09,4	06,8–12,8	44,1	42,6–45,7	
40–49	19,7	18,6–20,8	12,8	10,0–16,2	20,3	19,2–21,5	
50–59	17,0	15,9–17,9	23,6	20,1–27,6	16,3	15,5–17,4	
≥ 60	22,2	21,2–23,4	54,3	49,7–58,7	19,2	18,2–20,4	
Cor							0,288
Branca	47,8	46,4–49,3	48,4	43,9–53,0	47,8	46,2–49,3	
Preta	9,3	8,5–10,2	11,3	08,7–14,7	09,2	08,3–10,1	
Amarela e indígena	0,9	0,7–1,3	0,9	00,5–01,5	01,0	00,7–01,3	
Parda	42,0	40,5–43,3	39,4	35,2–43,6	42,1	40,7–43,6	
Nível de escolaridade							< 0,0001
Sem instrução até fundamental completo	49,3	47,8–50,7	67,2	62,7–71,5	47,6	46,1–49,1	
Médio incompleto e médio completo	33,8	32,4–35,2	23,3	19,4–27,6	34,8	33,3–36,3	
Superior incompleto e superior completo	16,9	15,8–18,1	09,5	07,2–12,5	17,7	16,4–18,9	
Plano de saúde							0,586
Sim	29,7	28,3–31,1	28,6	24,6–33,0	29,8	28,4–31,3	
Não	70,3	68,9–71,6	71,4	67,1–75,4	70,2	68,7–71,6	
Bolsa Família							0,427
Sim	9,4	8,7–10,1	08,4	06,1–11,4	09,5	08,8–10,3	
Não	90,6	89,8–91,3	91,6	88,6–93,9	90,5	89,7–91,2	
Autopercepção de saúde							< 0,0001
Boa e ótima	64,9	63,6–62,2	36,0	31,8–40,5	67,6	66,3–68,9	
Ruim, muito ruim e regular	35,1	33,8–36,4	64,0	59,5–68,2	32,4	31,1–33,8	
Região							0,019
Norte	6,9	6,6–7,3	05,1	04,3–06,2	07,1	06,7–07,5	
Nordeste	26,3	25,2–27,3	23,8	20,9–27,0	26,5	25,4–27,6	
Sudeste	44,3	42,8–45,8	49,3	44,8–53,8	43,8	42,2–45,5	
Sul	15,0	14,1–15,6	13,4	10,9–16,4	15,1	14,2–16,2	
Centro-Oeste	7,5	7,0–8,0	08,3	06,8–10,2	07,4	06,9–08,8	

IC95%: intervalo de confiança de 95%.

^a^Teste qui-quadrado de Pearson.

Foi estimado que 68,2% (IC95% 63,9–72,3) das pessoas com diabetes tinham conhecimento do seu diagnóstico, sendo que, destes, 92,2% (IC95% 88,6–94,7) estavam em tratamento medicamentoso. Foi estimado que 35,9% (IC95% 30,5–41,6) tinham níveis de HbA1c considerados normais (< 6,5%), e 48,1% (IC95%; 42,2–53,9) estavam com níveis de HbA1c abaixo de 7%, ou seja, com homeostase da glicose normalizada ou controlada entre os que receberam algum tipo de tratamento farmacológico ( Figura 2 ).

**Figura 2:** Prevalência e intervalos de confiança de 95% do conhecimento do diagnóstico, tratamento e controle do diabetes mellitus na população brasileira.

Nota: O conhecimento do diagnóstico foi definido pela proporção de participantes que responderam positivamente ao questionamento sobre terem recebido o diagnóstico de um profissional de saúde, ou que declararam utilizar medicação antidiabética. A proporção de participantes em tratamento foi obtida a partir da informação sobre o uso de medicamentos para diabetes ou insulina. O controle foi definido pela proporção de participantes que apresentaram valores de hemoglobina glicada (HbA1c) inferiores a 6,5%.

A proporção de conhecimento do diagnóstico de DM foi de 41,7% (IC95% 25,6–60,0) na faixa etária de 18 a 39 anos, 65,7% (IC95% 60,6–70,5) entre participantes sem plano de saúde, 47,5% (IC95% 31,6–64,0) no grupo que recebe o benefício Bolsa Família, 51,8% (IC95% 44,0–56,0) entre aqueles com boa/ótima autopercepção de saúde, e 51,5% (IC95% 42,9–60,0) em residentes da região Norte do país ( Tabela 2 ). Nesta mesma tabela observa-se que a proporção de tratamento medicamentoso foi menor nos indivíduos do sexo masculino (88%) (IC95% 80,0–93,1), na faixa etária de 40 a 49 anos (84,6%) (IC95% 61,6–94,9), e que não possuem plano de saúde (89,9%) (IC95% 85,0–93,4).

**Tabela 2:** Proporção do conhecimento do diagnóstico, tratamento e controle de diabetes mellitus na população adulta brasileira de acordo com as variáveis sociodemográficas (n = 8.435).

Características sociodemográficas	Conhecimento de diabetes mellitus			Tratamento de diabetes mellitus			Controle HbA1c < 6.5%			Controle HbA1c < 7.0%		

	%	IC95%	valor de p^a^	%	IC95%	valor de p^a^	%	IC95%	valor de p^a^	%	IC95%	valor de p^a^
Total	68,2	63,9–72,3		92,2	88,6–94,7		35,9	30,5–41,6		48,1	42,2–53,9	
Sexo			0,84			0,268			0,98			0,271
Masculino	67,6	60,6–74,0		88,0	80,0–93,1		35,7	26,8–45,7		43,6	33,9–53,8	
Feminino	68,6	63,0–73,7		94,7	91,3–96,8		35,9	29,4–42,9		50,6	43,5–57,7	
Idade (anos)			< 0,0001			0,421			0,005			0,002
18–39	41,7	25,6–60,0		94,0	76,9–98,7		14,2	05,4–32,7		14,2	05,4–32,7	
40–49	52,1	39,1–64,9		84,6	61,6–94,9		28,4	14,4–48,1		48,1	0,29–0,68	
50–59	71,6	63,1–78,8		92,5	84,7–96,5		25,3	16,3–37,1		38,3	0,26–0,50	
≥ 60	74,8	69,3–79,6		93,0	88,8–96,5		42,2	36,2–50,5		55,1	0,42–0,62	
Cor			0,117			0,919			0,7132			0,658
Branca	72,7	66,0–78,4		92,2	86,9–92,3		38,0	30,1–46,6		50,6	42,1–59,1	
Preta	60,8	46,3–73,6		90,9	76,2–96,9		34,0	18,7–53,4		48,3	30,1–66,9	
Amarela/indígena	48,2	23,1–74,3		100,0	–		40,3	12,1–76,8		40,3	12,1–76,8	
Parda	65,3	59,1–71,1		91,3	85,8–94,8		33,2	25,9–41,4		44,5	36,2–53,1	
Nível de escolaridade			0,668			0,547			0,735			0,848
Sem instrução até fundamental completo	68,2	63,0–73,0		91,1	86,3–94,3		35,3	29,1–42,1		48,9	42,2–55,7	
Médio incompleto e médio completo	66,1	56,1–74,8		93,9	85,1–97,6		34,2	22,6–48,1		44,8	31,7–58,6	
Superior incompleto e superior completo	73,4	59,8–83,6		95,5	82,2–99,0		42,4	26,4–60,2		49,6	32,1–67,3	
Plano de saúde			0,079			0,015			0,976			0,79
Sim	74,3	65,9–81,2		96,9	92,3–98,8		35,7	26,7–45,8		46,9	36,8–57,2	
Não	65,7	60,6–70,5		89,9	85,0–93,4		35,9	29,4–42,9		48,6	41,6–55,7	
Bolsa Família			0,006			0,221			0,028			0,41
Sim	47,5	31,6–64,0		96,8	85,9–99,3		17,6	07,9–34,5		38,5	19,3–62,1	
Não	70,1	65,7–74,2		91,9	88,1–94,5		37,0	31,4–43,0		48,7	42,7–54,7	
Autopercepção de saúde			< 0,0001			0,364			0,002			0,0077
Boa e ótima	51,8	44,0–56,0		90,0	82,6–94,5		50,8	39,3–62,1		61,6	49,7–72,2	
Ruim, muito ruim e regular	77,4	72,7–81,5		93,0	88,5–95,8		30,4	24,7–36,9		43,1	36,6–49,5	
Região			0,004			0,864			0,71			0,9052
Norte	51,5	42,9–60,0		92,8	83,6–97,0		37,3	26,1–49,9		47,1	35,1–59,5	
Nordeste	59,2	52,7–65,4		92,1	86,6–95,5		40,0	32,0–48,6		50,8	42,3–59,3	
Sudeste	73,8	66,2–80,2		92,9	86,4–96,4		35,7	27,0–44,7		47,5	38,3–56,9	
Sul	67,2	56,1–76,7		90,9	79,5–96,2		30,0	19,0–43,9		44,5	31,4–58,4	
Centro-Oeste	71,3	60,9–79,8		89,1	78,6–94,8		37,1	25,9–49,8		50,5	37,7–63,2	

IC95%: intervalo de confiança de 95%; HbA1c: hemoglobina glicada.

^a^Teste qui-quadrado de Pearson.

Ainda na Tabela 2 , são mostradas as proporções de portadores de DM que estão sob controle usando dois pontos de corte: HbA1c < 6,5% foi de 35,8%, e HbA1c < 7% foi de 48,1%. Observa-se, ainda, um menor controle, a partir do primeiro ponto de corte, nos indivíduos do sexo masculino (35,7%) (IC95% 26,8–45,7) e que declaram autopercepção de saúde ruim (30,4%) (IC95% 24,7–36,9). Com relação ao segundo ponto de corte, observa-se um menor controle em indivíduos do sexo masculino (43,6%) (IC95% 33,9–53,8), escolaridade com ensino médio incompleto/médio completo (44,8%) (IC95% 31,7–58,6) e que relataram autopercepção de saúde ruim (43,1%) (IC95% 36,6–49,5).

## DISCUSSÃO

Neste estudo foi estimado que aproximadamente um a cada dez brasileiros tem diagnóstico de diabetes, e destes, 68,2% têm conhecimento do diagnóstico. Além disso, a maioria dos diabéticos encontra-se sob tratamento medicamentoso, e menos da metade desses tem seus níveis de HbA1c inferiores a 6,5%, ou seja, controlados. Ao considerar metas de controle glicêmico mais flexíveis, observou-se que 48,1% da população adulta têm HbA1c < 7%, e 65,7% dos idosos têm HbA1c < 7,5%.

As mais altas prevalências de DM foram no grupo acima de 60 anos, que se declararam da raça/cor amarela/indígena, de baixa escolaridade, com autopercepção de saúde ruim, e nas populações do Centro–Oeste e Sudeste brasileiros. Baixas proporções de conhecimento, controle e tratamento foram mais frequentes em homens, com idade de 18 a 39 anos, indivíduos que possuem baixa escolaridade, sem plano de saúde e beneficiários do Programa Bolsa Família.

As altas proporções de participantes que desconhecem seu diagnóstico neste estudo se concentram nos grupos de baixos níveis socioeconômicos, participantes do programa Bolsa Família, e residentes da região Norte do país, como já observados em outro estudo^16^.

Um aspecto adicional, que foi mostrado em estudo recente, observou que populações com poucos recursos socioeconômicos também apresentam altas prevalências de tabagismo, excesso de peso e obesidade, baixo consumo de frutas e hortaliças e alto consumo de bebidas adoçadas, e, nas mulheres, baixo acesso a programas de rastreamento de câncer de colo e mama^16^.

Estratégias como a Campanha Nacional da Detecção de DM, a qual atingiu mais de 20 milhões de usuários do Sistema Único de Saúde (SUS) com idade superior a 40 anos^17^, devem ser reeditadas. Porém, estratégias de uso de tecnologia leve do cuidado, de baixo custo e ampla acessibilidade da população brasileira podem ser necessárias.

A população brasileira certamente não está longe das estimativas de prevalências globais. Recente estudo apresenta que aproximadamente 50% dos diabéticos adultos não sabem do seu diagnóstico, e que, destes, 84,3% estão residindo em países em desenvolvimento^18^. Uma alternativa de baixo custo, não invasiva e de fácil aplicação, seria o uso do Escore Finlandês de Risco de Diabetes (FINDRISC), o qual mede o risco de desenvolver DM2 em adultos. Essa estratégia tem sua validade demonstrada em nível nacional^19^.

Baixas prevalências de controle do diabetes aferido pelos níveis de hemoglobina glicada (< 6,5%) foram mostrados nos grupos de baixa escolaridade e beneficiários do Programa Bolsa Família. Esses achados reforçam a vulnerabilidade social e o acesso a um controle eficaz das doenças, corroborando achados de outros estudos^20^.

Estimativas usando dados da PNS 2019 demonstraram que os mais jovens faziam menor consumo de medicação^3^, o que pode ser justificado pela menor gravidade da patologia, sendo possível favorecer o manejo por meio de medidas não farmacológicas, como o incentivo à atividade física e a alimentação saudável. Além disso, o DM do tipo 1 (DM1), não identificado neste estudo, pode apresentar menor controle glicêmico, em decorrência da gravidade da doença, bem como a resistência ao uso de medicamentos^21^.

Também foi possível identificar uma maior frequência de internação nos jovens de 18 a 29 anos, o que pode ser compreendido pela maior prevalência de DM1 em adultos jovens, em função dos sintomas agudos da doença e, ainda, da não adaptação aos novos cuidados e da menor adesão às práticas de cuidadores^4^. Também identificaram menor internação por causa da DM ou de alguma complicação entre as mulheres, o que pode significar melhor controle entre elas, dado que coincide com os achados do presente estudo^4^.

A alta proporção de pessoas com diabetes em tratamento estimada neste estudo responde às políticas públicas implantadas para a melhoria do acesso à medicação, por meio dos programas Farmácia Popular e Saúde Não Tem Preço, o que possibilita acesso gratuito desses remédios nas Unidades Básicas de Saúde e nas farmácias credenciadas ao programa. Os sistemas de vigilância também evidenciaram que 70,3% das pessoas diagnosticadas com DM obtiveram remédios orais gratuitos através da farmácia do SUS ou do Programa Farmácia Popular, e que 90% tiveram acesso grátis à insulina^22^. Destacam-se também os resultados obtidos pela Pesquisa Nacional sobre Acesso, Utilização e Promoção do Uso Racional de Medicamentos (PNAUM), um inquérito domiciliar de base populacional, que mostrou que 21,4% obtiveram os medicamentos para diabetes de forma paga, e 78,6% de forma gratuita^23^. Ainda confirmando estas conclusões, 57,4% relataram na PNS 2013 terem obtido fármacos para diabetes no Programa Farmácia Popular^24^. Neste estudo, não temos informações para esclarecer se a prevalência de participantes sem tratamento está relacionada com a escolha de medicamentos ou se seria em decorrência da dificuldade de acesso^25^.

O acompanhamento dos pacientes com diabetes após o diagnóstico, na APS, inclui a realização da consulta médica, de enfermagem e participação em grupos de diabéticos, e o quantitativo de consultas com cada profissional sofre alteração em decorrência da condição clínica do paciente^26^. Destaca-se também a necessidade de realizar exame de glicemia de jejum e HbA1c duas vezes ao ano, em indivíduos que estão sobre controle glicêmico, e a cada três meses para aqueles que não estão^26^.

A manutenção dos níveis de glicose dentro da normalidade é fundamental para o enfrentamento do DM. Neste estudo, a prevalência de controle foi baixa, não muito longe de achados em outras populações, como as de Curdistão iraniano (18,30%)^27^, Coreia (42,5%)^28^e Mianmar (40,8%)^29^. Estudos anteriores estimaram em 26% e 78% a prevalência de controle de níveis glicêmicos em população atendida em serviços públicos de saúde e serviços privados, respectivamente, porém trata-se de estudos locais e sem representatividade nacional, o que dificulta a sua comparação com o presente trabalho^12 , 13^.

Falhas no controle glicêmico podem contribuir para elevar o risco de doenças cardiovasculares, nefropatias, neuropatias, retinopatias, bem como aumento de hospitalizações^30^. Destaca-se que a complexidade inerente às terapias medicamentosas contribui para elevar o risco quanto ao erro de medicação, e necessita que o usuário detenha habilidades para cumprir os cuidados dispensados pelos profissionais da saúde^31 , 32^. O desafio da terapia medicamentosa foi acompanhado pelo quantitativo de medicamentos em uso, decorrente dos pacientes que apresentam alta complexidade e faziam uso de polifarmácia^33^.O empoderamento do paciente no processo de autocuidado, a educação em saúde, especialmente quanto às informações referentes ao horário de medicação, e o uso correto dos fármacos em conformidade com a prescrição médica, são estratégias necessárias para atingir o controle da doença^34^.

O controle glicêmico é fundamental para diminuir o risco das complicações do DM e de doença cardiovascular. Deve-se atentar também para o monitoramento do perfil lipídico, e deve-se dar tratamento adequado para atingir o controle glicêmico. O manejo do DM na atenção primária segue estratégia do incentivo de hábitos de vida saudáveis. Porém, na prática, o programa tem foco basicamente na oferta de medicamentos. Esse processo de trabalho dificulta o alcance dos objetivos, como reconhecimento e dimensionamento dos problemas de saúde, tanto no âmbito individual quanto coletivo, os quais auxiliam de forma mais eficaz nas ações/intervenções de saúde, como mostrado em um estudo desenvolvido no munícipio de São Paulo^35^. Estudos mostram que não existe vínculo entre os pacientes portadores de DM e ações dos profissionais de saúde, corroborando para descontinuidade da adesão ao tratamento e impactando diretamente no seu controle^36^.

Os resultados do baixo controle do DM identificados neste estudo podem ser explicados pela complexidade do manejo da doença, que perpassa pelo monitoramento dos valores glicêmicos, adesão ao tratamento, bem como pela inclusão de prática de atividade física regular e alteração na dieta^37^. Nesta perspectiva, as estratégias que contribuem para o empoderamento do paciente podem auxiliar no processo de desenvolvimento de adoção de novas atitudes e habilidades, que irão promover alterações nos hábitos/estilo de vida e, consequentemente, no autocuidado. Ensaios clínicos utilizando estratégias de educação por meio de intervenção telefônica, programa de capacitação, e visita domiciliar, mostraram que essas intervenções têm contribuído positivamente para melhoria dos resultados dos níveis de HbA1c, bem como para o empoderamento e autocuidado^38^. As visitas domiciliares realizadas pelos agentes comunitários de saúde (ACS) contribuem para o controle e supervisão do tratamento, melhorando a adesão às práticas de autocuidado referentes à DM2^39^.

Ressalta–se que a magnitude de agravos mostrados pode piorar em decorrência do novo modelo de financiamento da APS no SUS^40^, denominado “Previne Brasil”, pois fortalece apenas as estratégias biomédicas, o que poderia acarretar atrasos na detecção do DM e piora e diminuição da prevalência de controle. Além disso, vale destacar que os efeitos gerados pela pandemia do covid–19 poderão, em curto prazo, afetar o desempenho do cuidado dispensado às doenças crônicas, piorando os níveis de controle e detecção da doença^41^.

Uma limitação deste estudo refere–se à falta de dados referentes à medicação utilizada, seu tempo de uso e adesão ao tratamento, que se limitou a registrar apenas o uso de antidiabéticos e/ou injeções de insulina. Medidas não farmacológicas são também necessárias ao controle glicêmico, e não foram avaliadas neste estudo, por exemplo. Esses dados possibilitariam um melhor conhecimento do baixo desempenho no controle dos níveis glicêmicos encontrados na população brasileira, apesar do amplo acesso aos medicamentos e ao tratamento. Contudo, a utilização de dados com base populacional e representatividade nacional configura uma adequada validade externa.

Destaca-se também a medida dos níveis de HbA1c colhida de sangue venoso e não capilar, considerada padrão ouro para detecção da doença. A ausência de consenso para definição do ponto de corte da HbA1c no controle da doença contribui para dificultar o processo de avaliação e monitoramento do DM. Sendo assim, este estudo optou por trabalhar com os dois pontos de corte.

Registra-se ainda, como limitação, a não distinção entre o tipo de DM (DM1 ou DM2). Ressalta-se que este é um estudo populacional inédito que utiliza dados laboratoriais com representatividade da população brasileira para estimar o conhecimento, tratamento e controle do DM, aspectos fundamentais para contribuir com os programas de saúde pública no enfrentamento da doença.

A proporção de conhecimento do DM na população brasileira foi estimada em 68%, e, destes, 92%, estavam em tratamento medicamentoso. O controle adequado foi estimado em 36%, considerando o critério mais rigoroso (HbA1c < 6,5%). Alguns subgrupos populacionais, tais como os que não possuem plano de saúde, os que relataram ter autopercepção de saúde ruim e os beneficiários do Programa Bolsa Família, apresentaram pior desempenho do conhecimento sobre a doença. Os dados deste estudo podem contribuir para o fortalecimento de políticas públicas que objetivam promover o estilo de vida saudável. A implementação de estratégias inovadoras para auxiliar no controle do DM é fundamental para enfrentar a carga de doença atribuída ao DM no Brasil.
